# SARS-CoV-2 infection induces long-lived bone marrow plasma cells in humans

**DOI:** 10.21203/rs.3.rs-132821/v1

**Published:** 2020-12-31

**Authors:** Jackson S. Turner, Wooseob Kim, Elizaveta Kalaidina, Charles W. Goss, Adriana M. Rauseo, Aaron J. Schmitz, Lena Hansen, Alem Haile, Michael K. Klebert, Iskra Pusic, Jane A. O’Halloran, Rachel M. Presti, Ali H. Ellebedy

**Affiliations:** 1 Department of Pathology and Immunology, Washington University School of Medicine, St. Louis, MO, USA; 2 Division of Allergy and Immunology, Department of Internal Medicine, Washington University School of Medicine, St. Louis, MO, USA; 3 Division of Biostatistics, Washington University School of Medicine, St. Louis, MO, USA; 4 Division of Infectious Diseases, Department of lnternal Medicine, Washington University School of Medicine, St. Louis, MO, USA; 5 Influenza Centre, Department of Clinical Science, University of Bergen, 5021 Bergen, Norway; 6 Clinical Trials Unit, Washington University School of Medicine, St. Louis, MO, USA; 7 Division of Oncology, Department of Internal Medicine, Washington University School of Medicine, St. Louis, MO, USA; 8 The Andrew M. and Jane M. Bursky Center for Human Immunology & Immunotherapy Programs

## Abstract

Infection or vaccination induces a population of long-lived bone marrow plasma cells (BMPCs) that are a persistent and essential source of protective antibodies^1–5^. Whether this population is induced in patients infected with the severe acute respiratory syndrome coronavirus 2 (SARS-CoV-2) is unknown. Recent reports have suggested that SARS-CoV-2 convalescent patients experience a rapid decay in their antigen-specific serum antibodies, raising concerns that humoral immunity against this virus may be short-lived^6–8^. Here we show that in patients who experienced mild infections (n=73), serum anti-SARS-CoV-2 spike (S) antibodies indeed decline rapidly in the first 3 to 4 months after infection. However, this is followed by a more stable phase between 4- and 8-months after infection with a slower serum anti-S antibody decay rate. The level of serum antibodies correlated with the frequency of S-specific long-lived BMPCs obtained from 18 SARS-CoV-2 convalescent patients 7 to 8 months after infection. S-specific BMPCs were not detected in aspirates from 11 healthy subjects with no history of SARS-CoV-2 infection. Comparable frequencies of BMPCs specific to contemporary influenza virus antigens or tetanus and diphtheria vaccine antigens were present in aspirates in both groups. Circulating memory B cells (MBCs) directed against the S protein were detected in the SARS-CoV-2 convalescent patients but not in uninfected controls, whereas both groups had MBCs against influenza virus hemagglutinin. Overall, we show that robust antigen specific long-lived BMPCs and MBCs are induced after mild SARS-CoV-2 infection of humans.

## Introduction

Reinfections by seasonal coronaviruses occur 6–12 months after the previous infection, indicating that protective immunity against these viruses may be short-lived^9,10^. Early reports documenting rapidly declining antibody titers in convalescent SARS-CoV-2 patients in the first several months after infection suggested that protective immunity against SARS-CoV-2 may be similarly transient^6–8^. It was recently suggested that SARS-CoV-2 infection may fail to elicit a functional germinal center response, interfering with generation of the long-lived plasma cells that maintain durable antibody titers^2–5,11^. However, more recent reports analyzing samples collected approximately 4 to 6 months after infection indicate that SARS-CoV-2 antibody titers decline more slowly^12–15^. Because durable serum antibody titers are maintained by long-lived bone marrow plasma cells (BMPCs), we sought to determine whether they were detectable in SARS-CoV-2 convalescent patients approximately 7 months after infection.

## Results

### Biphasic decline in serum SARS-CoV-2 antibody titers

**Figure 1.**
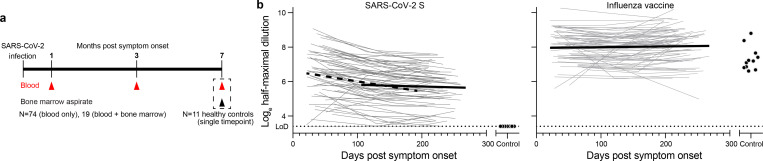
Biphasic decline in SARS-CoV-2 antibody titers a) Study design. Seventy-three SARS-CoV-2 convalescent patients with mild disease (ages 21–69) were enrolled and blood was collected approximately 1 month, 4 months, and 7 months post onset of symptoms. Bone marrow aspirates were collected from eighteen of the participants 7 to 8 months after infection and from eleven healthy volunteers (ages 23–60) with no history of SARS-CoV-2 infection. b) Blood IgG titers against S (left) and influenza virus vaccine (right) measured by ELISA in convalescent patients (open circles) at the indicated time post onset of symptoms and controls (closed circles). Dotted line indicates limit of detection. Heavy lines represent modeled antibody decay kinetics for S titers 1–4 months (dashed line; decay rate −0.00595, *P*<0.001, t_1/2_=116.5 days) and 4–7 months (solid line; decay rate −0.00101, *P*=0.04, t_1/2_=686.3 days) post symptom onset and for influenza virus vaccine titers 1–7 months post symptom onset (decay rate 0.000455, *P*=0.33) estimated using a longitudinal linear mixed model approach (see Methods for details).

Blood samples were collected approximately 1 month after onset of symptoms from seventy-three SARS-CoV-2 convalescent volunteers (47.9% female, 52.1% male, median age 49), the majority of whom had experienced mild illness (8.3% hospitalized, Extended Data Table 1). Follow-up blood samples were collected twice at 3-month intervals. Additionally, bone marrow aspirates were collected from eighteen of the participants 7 to 8 months after infection and from eleven healthy volunteers with no history of SARS-CoV-2 infection (Fig. 1a, Extended Data Table 2). We first performed a longitudinal analysis of circulating anti-SARS-CoV-2 serum antibodies. While anti-SARS-CoV-2 spike (S) IgG antibodies were undetectable in blood from controls, 70 of 73 convalescent participants had detectable serum titers approximately 1 month after onset of symptoms. Between 1- and 3-months post symptom onset, anti-S IgG titers declined with an estimated half-life of 116.5 days. However, in the interval between 3- and 8-months post symptom onset, the decay rate slowed, and titers waned with an estimated half-life of 686.3 days. In contrast, IgG titers against the 2019/2020 inactivated seasonal influenza virus vaccine were detected in all control and SARS-CoV-2 convalescent participants and were stable over the course of the study (Fig. 1b).

### SARS-CoV-2 infection elicits long-lived BMPCs

**Figure 2.**
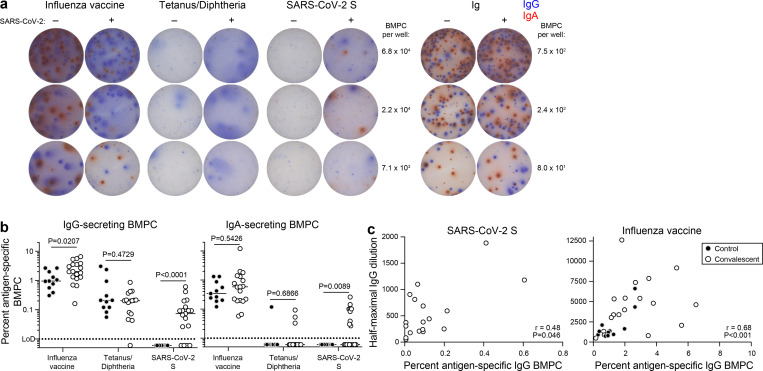
SARS-CoV-2 infection elicits long-lived BMPCs a) Example images of ELISpot wells coated with the indicated antigens or anti-Ig and developed in blue and red for IgG and IgA, respectively after incubation of magnetically enriched BMPC from convalescent and control participants. b) Frequencies of BMPC secreting IgG (left) or IgA (right) antibodies specific for the indicated antigens, indicated as percentages of total IgG- or IgA-secreting BMPC in convalescent (open circles) and control (closed circles) participants. Horizontal lines indicate medians. *P*-values from two-sided Mann-Whitney U-tests. c) Frequencies of IgG BMPC specific for S (left) and influenza virus vaccine (right) plotted against respective IgG titers in paired blood samples from convalescent patients 7 months post symptom onset (open circles) and control participants (closed circles). Each symbol represents one sample (n=18 convalescent, 11 control). *P*- and *r*-values from two-sided Spearman’s correlations.

The biphasic decay in anti-S IgG is consistent with a transition of serum antibody titers from being supported by short-lived plasmablasts to a smaller but more persistent population of long-lived plasma cells generated later in the immune response. The majority of this latter population resides in bone marrow^1,2^. To investigate whether SARS-CoV-2 convalescent patients developed a virus specific long-lived BMPC compartment, we examined their bone marrow aspirates obtained 7 to 8 months after infection for anti-SARS-CoV-2 S-specific long-lived BMPCs. BMPCs were magnetically enriched from the aspirates and we then quantified the frequencies of those secreting IgG and IgA directed against the 2019/2020 influenza virus vaccine, tetanus/diphtheria vaccine, and SARS-CoV-2 S protein by ELISpot (Fig. 2a). Frequencies of influenza and tetanus/diphtheria vaccine specific BMPCs were comparable between control and convalescent participants. IgG- and IgA-secreting S-specific BMPCs were detected in 14 and 9 of the 18 convalescent participants, respectively, but not in any of the 11 control participants (Fig. 2b). Importantly, none of the convalescent patients had detectable plasmablasts in blood at the time of bone marrow sampling, indicating that the detected BMPCs represent long-lived, bone marrow resident cells and were not recently generated. Frequencies of anti-S IgG BMPCs showed a modest but significant correlation with circulating IgG titers 7–8 months post symptom onset in convalescent participants, consistent with long-term maintenance of antibody levels by these cells. In accordance with previous reports^16–18^, frequencies of influenza vaccine-specific IgG BMPCs and antibody titers exhibited a strong and significant correlation (Fig. 2c).

### SARS-CoV-2 infection elicits a robust memory B cell response

**Extended Data Figure 1.**
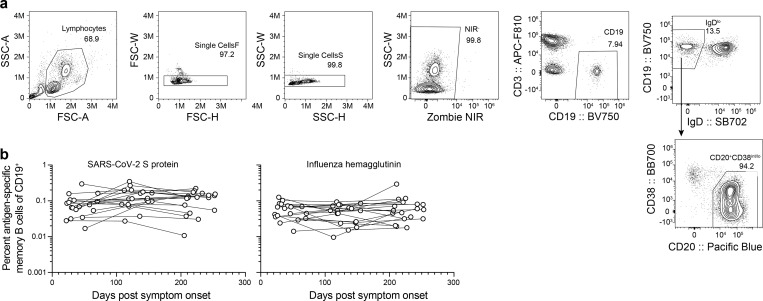
SARS-CoV-2 memory B cells. a) Flow cytometry gating strategy for isotype-switched memory B cells in PBMC. (b) Kinetics of S- (left) and influenza virus hemagglutinin- (right) binding memory B cells, gated as in (a) and Fig. 3a, in PBMCs from convalescent patients collected at the indicated days post onset of symptoms. Symbols at each timepoint represent one sample (n=18). Data from the 7-month timepoint are also shown in Fig. 3b.

**Figure 3.**
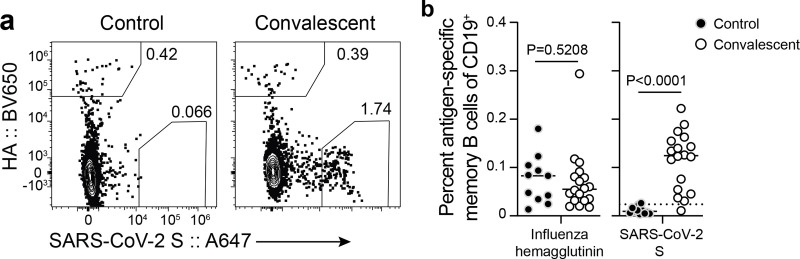
SARS-CoV-2 infection elicits a robust memory B cell response a) Example plots of influenza virus hemagglutinin (HA) and S staining on IgD^lo^ CD20^+^ CD38^lo/int^ CD19^+^ CD3^−^ live singlet lymphocytes (gating in Extended Data Fig. 1a) from control (left) and convalescent (right) PBMC 7 months after symptom onset. b) Frequencies of influenza virus HA- (left) and S- (right) binding memory B cells in PBMC from control (closed circles) and convalescent (open circles) participants 7 months after symptom onset. The dotted line in the S plot indicates limit of sensitivity, defined as the median + 2× SD of the controls. Each symbol represents one sample (n=18 convalescent, 11 control). Horizontal lines indicate medians. *P*-values from two-sided Mann-Whitney U-tests.

Memory B cells (MBCs) form the other component of humoral immune memory. Upon antigen re-exposure, MBCs rapidly expand and differentiate into antibody-secreting plasmablasts. We examined the frequency of SARS-CoV-2 specific circulating MBC pool in the convalescent patients as well as in the healthy controls. We stained peripheral blood mononuclear cells with fluorescently labeled S probes and determined the frequency of S-binding MBCs among isotype-switched IgD^lo^ CD20^+^ MBCs by flow cytometry. For comparison, we also co-stained the cells with fluorescently labeled influenza virus hemagglutinin (HA) probes (Fig. 2a, Extended Data Fig. 1a). S-binding MBCs were identified in convalescent patients in the first sample collected approximately 1 month after onset of symptoms, with comparable frequencies to influenza HA-binding memory B cells (Extended Data Fig. 1b). S-binding memory B cells were maintained through the end of the study and were present at significantly higher frequencies compared to healthy controls, comparable to frequencies of influenza HA-binding memory B cells identified in both groups (Fig. 3b).

## Discussion

This study sought to determine whether SARS-CoV-2 infection induced antigen-specific long-lived BMPCs in humans. We detected SARS-CoV-2 S-specific BMPCs in aspirates from 14 of 18 convalescent patients, and in none from the 11 control participants. Frequencies of anti-S IgG BMPCs modestly correlated with serum IgG titers 7–8 months after infection. Finally, we showed that S-binding MBCs in blood of convalescent patients are present at similar frequencies as those directed against influenza virus HA. Altogether, our results are consistent with SARS-CoV-2 infection eliciting a canonical T-dependent B cell response, in which an early transient burst of extrafollicular plasmablasts generates an early wave of serum antibodies that decline relatively quickly once a peak titer is reached. This is followed by more stably maintained serum antibody levels that are supported by long-lived BMPCs. Our data suggest that SARS-CoV-2 infection induces a germinal center response in humans because long-lived BMPCs are thought to be predominantly germinal center-derived^5^. This is consistent with recent data showing increased levels of somatic hypermutation in MBCs targeting the receptor binding domain of the S protein in SARS-CoV-2 convalescent patients at 6 months compared to 1 month after infection^15^.

To our knowledge, the current study provides the first direct evidence for induction of antigen specific BMPCs after a viral infection in humans, but it does have some limitations. Although we detected anti-S IgG antibodies in serum 7 months after infection in all 18 of the convalescent donors from whom we obtained bone marrow aspirates, we failed to detect S-specific BMPCs in four donors. Serum anti-S antibody titers in those four donors were low, suggesting that S-specific BMPCs may potentially be present at very low frequencies that are below our limit of detection. Another limitation is while SARS-CoV-2 S protein is the main target of neutralizing antibodies^12,19–24^, we do not know the fraction of the S-specific BMPCs detected 7 months after infection in our study encoding neutralizing antibodies. It is important to note, however, that correlation between serum anti-S IgG binding and neutralization titers has been documented^12,25^. Further studies will be required to determine the epitopes targeted by BMPCs and MBCs and how preexisting immunity to human coronaviruses could alter the immune response to SARS-CoV-2 infection and immunization. Finally, while our data document a robust induction of long-lived BMPCs after SARS-CoV-2 infection, it is critical to note that our convalescent patients mostly experienced mild infections. Our data are consistent with a recent report showing that individuals who recovered rapidly from symptomatic SARS-CoV-2 infection generated a robust humoral immune response^26^. Therefore, it is possible that more severe SARS-CoV-2 infections could lead to a different outcome with respect to long-lived BMPC frequencies due to dysregulated humoral immune responses. This, however, has not been the case in survivors of the 2014 West African Ebola virus outbreak in whom severe viral infection induced long-lasting antigen-specific serum IgG antibodies^27^.

Long-lived BMPCs provide the host with a persistent source of preformed protective antibodies and are therefore needed to maintain durable immune protection. However, longevity of serum anti-S IgG antibodies is not the only determinant of how durable immune-mediated protection will be. Indeed, isotype-switched MBCs can rapidly differentiate into antibody secreting cells upon pathogen reexposure, offering a second line of defense^28^. Encouragingly, the frequency of S-binding circulating MBCs 7-months after infection was higher than those directed against contemporary influenza HA antigens. These data indicate that even at that late time point, more MBC and BMPC precursors may still be emerging from ongoing germinal center reactions. We have recently shown that germinal center reactions can persist for at least two months in the draining lymph nodes after non-adjuvanted seasonal influenza virus vaccination^29^. It is not unreasonable to assume that SARS-CoV-2 infection would foster a more persistent germinal center reaction, but future studies will be needed to precisely determine the dynamics of such responses. Overall, our data provide strong evidence that SARS-CoV-2 infection in humans robustly establishes the two arms of humoral immune memory: long-lived BMPC and MBCs. These findings provide an immunogenicity benchmark for SARS-CoV-2 vaccines and a foundation for assessing the durability of primary humoral immune responses induced after viral infections in humans.

## Methods

### Sample collection, preparation, and storage.

All studies were approved by the Institutional Review Board of Washington University in St. Louis. Written consent was obtained from all participants. Seventy-four participants who had recovered from SARS-CoV-2 infection and eleven controls without SARS-CoV-2 infection history were enrolled (Extended Data Table 1). Blood samples were collected in EDTA tubes and peripheral blood mononuclear cells (PBMCs) were enriched by density gradient centrifugation over Ficoll 1077 (GE) or Lymphopure (BioLegend), remaining red blood cells were lysed with ammonium chloride lysis buffer, and cells were immediately used or cryopreserved in 10% dimethylsulfoxide in FBS. Approximately 30 mL bone marrow aspirates were collected in EDTA tubes from the iliac crest of eighteen convalescent participants and the controls. Bone marrow mononuclear cells were enriched by density gradient centrifugation over Ficoll 1077, remaining red blood cells were lysed with ammonium chloride buffer (Lonza) and washed with PBS supplemented with 2% FBS and 2 mM EDTA. Bone marrow plasma cells were enriched from bone marrow mononuclear cells using CD138 Positive Selection Kit II (Stemcell) and immediately used for ELISpot.

### Antigens.

For ELISpot, plates were coated with Flucelvax Quadrivalent 2019/2020 seasonal influenza virus vaccine (Sequiris), tetanus/diphtheria vaccine (Grifols), or recombinant soluble Spike protein derived from SARS-CoV-2, expressed as previously described^30^. Briefly, mammalian cell codon-optimized nucleotide sequences coding for the soluble version of the spike protein of SARS-CoV-2 (GenBank: MN908947.3, amino acids 1–1213) including a C-terminal thrombin cleavage site, T4 foldon trimerization domain, and hexahistidine tag cloned into mammalian expression vector pCAGGS. The spike protein sequence was modified to remove the polybasic cleavage site (RRAR to A) and two stabilizing mutations were introduced (K986P and V987P, wild type numbering). Recombinant proteins were produced in Expi293F cells (ThermoFisher) by transfection with purified DNA using the ExpiFectamine 293 Transfection Kit (ThermoFisher). Supernatants from transfected cells were harvested 3 days post-transfection, and recombinant proteins were purified using Ni-NTA agarose (ThermoFisher), then buffer exchanged into phosphate buffered saline (PBS) and concentrated using Amicon Ultracel centrifugal filters (EMD Millipore).For flow cytometry staining, recombinant S was labeled with Alexa Fluor 647-NHS ester (Thermo Fisher). Recombinant HA from A/Brisbane/02/2018 (a.a.18–529) and B/Colorado/06/2017 (a.a. 18–546) expressed in 293 cells were purchased from Immune Technology and biotinylated using the EZ-Link Micro NHS-PEG4-Biotinylation Kit (Thermo Fisher); excess biotin and Alexa Fluor 647 were removed using 7-kDa Zeba desalting columns (Pierce).

### ELISpot.

Direct *ex-vivo* ELISpot was performed to determine the number of total, vaccine-binding, or recombinant Spike-binding IgG- and IgA-secreting cells present in BMPC and PBMC samples using IgG/IgA double-color ELISpot Kits (Cellular Technologies, Ltd.) according to the manufacturer’s instructions. ELISpot plates were analyzed using an ELISpot counter (Cellular Technologies Ltd.).

### ELISA.

Assays were performed in 96-well plates (MaxiSorp; Thermo) coated with 100 μL of Flucelvax 2019/2020 or recombinant Spike protein in PBS, and plates were incubated at 4 °C overnight. Plates were then blocked with 10% FBS and 0.05% Tween20 in PBS. Serum or plasma were serially diluted in blocking buffer and added to the plates. Plates were incubated for 90 min at room temperature and then washed 3 times with 0.05% Tween-20 in PBS. Goat anti-human IgG-HRP (Jackson ImmunoResearch, 1:2,500) was diluted in blocking buffer before adding to wells and incubating for 60 min at room temperature. Plates were washed 3 times with 0.05% Tween20 in PBS, and then washed 3 times with PBS before the addition of peroxidase substrate (SigmaFAST o-Phenylenediamine dihydrochloride, Sigma-Aldrich). Reactions were stopped by the addition of 1 M HCl. Optical density measurements were taken at 490 nm. The half-maximal binding dilution for each serum or plasma sample was calculated using nonlinear regression (Graphpad Prism v8). The limit of detection was defined as 1:30.

### Statistics.

Spearman’s correlation coefficients were estimated to assess the relationship between 7-month anti-S and anti-influenza virus vaccine IgG titers and frequencies of BMPCs secreting IgG specific for S and influenza virus vaccine, respectively. Decay rates based on the log-transformed S and influenza virus vaccine titers were estimated using a longitudinal linear mixed model approach. Time since symptom onset (days) was included as a fixed effect in these models and subject-specific random intercepts and slopes were included to adjust for repeated measurements. Exponential decay rate was estimated as the slope for the fixed effect and the half-life was estimated as ln(0.5)/decay rate. In addition to the analysis of all time points in a single model, we assessed whether there was evidence of a change in decay rate over the course of observation for anti-S titer by separately analyzing only the first and second measurements, and only the second and third measurements. These models only included 2 time points per person, and we removed the random slope parameter from these models. This resulted in a single mixed model for the anti-influenza virus vaccine titer, and three separate models for the anti-S titer. All analyses were conducted using SAS 9.4 (SAS Institute Inc, Cary, NC, USA) and Prism 8.4 (Graphpad), and *P*-values < 0.05 were considered significant.

### Flow cytometry.

Staining for flow cytometry analysis was performed using cryopreserved PBMCs. Cells were stained for 30 min on ice with biotinylated recombinant HAs diluted in in 2% FBS and 2 mM EDTA in PBS (P2), washed twice, then stained for 30 min on ice with Alexa 647-conjugated S, IgA-FITC (M24A, Millipore, 1:500), IgG-BV480 (goat polyclonal, Jackson ImmunoResearch, 1:100), IgD-SB702 (IA6–2, Thermo, 1:50), CD38-BB700 (HIT2, BD Horizon, 1:500), CD20-Pacific Blue (2H7, 1:400), CD4-BV570 (OKT4, 1:50), CD24-BV605 (ML5, 1:100), streptavidin-BV650, CD19-BV750 (HIB19, 1:100), CD71-PE (CY1G4, 1:400), CXCR5-PE-Dazzle 594 (J252D4, 1:50), CD27-PE-Cy7 (O323, 1:200), IgM-APC-Fire750 (MHM-88, 1:100), CD3-APC-Fire810 (SK7, 1:500), and Zombie NIR (all BioLegend) diluted in Brilliant Staining buffer (BD Horizon). Cells were washed twice with P2 and acquired on an Aurora using SpectroFlo v2.2 (Cytek). Flow cytometry data were analyzed using FlowJo v10 (Treestar). In each experiment, PBMC were included from convalescent and control participants.

## Extended Data

**Extended Data Table 1.** Convalescent patient characteristics

Variable	Total N=73 N (%)

**Age (median [range])**	49 (21–69)

**Sex**	

Female	35 (47.9)

Male	38 (52.1)

**Race**	

White	66 (90.4)

Black	1 (1.4)

Asian	4 (5.5)

Other	2 (2.7)

**Comorbidities**	

Asthma	12 (16.2)

Lung disease	0 (0)

Heart disease	3 (4.1)

Hypertension	13 (17.6)

Diabetes mellitus	3 (4.1)

Cancer	10 (13.5)

Autoimmune disease	4 (5.4)

Hyperlipidemia	8 (10.8)

Hypothyroidism	5 (6.8)

Gastroesophageal reflux disease	5 (6.8)

Other	26 (35.1)

*Obesity*	1 (1.4)

*Solid organ transplant*	1 (1.4)

**First symptom**	

Cough	12 (16.4)

Diarrhea	1 (1.4)

Dyspnea	2 (2.7)

Fatigue	6 (8.2)

Fever	22 (30.1)

Headache	8 (11)

Loss of taste	3 (4.1)

Malaise	3 (4.1)

Myalgias	9 (12.3)

Nasal congestion	2 (2.7)

Nausea	1 (1.4)

Night sweats	1 (1.4)

Sore throat	3 (4.1)

**Symptom present during disease**	

Fever	62 (84.9)

Cough	51 (69.9)

Dyspnea	28 (38.4)

Nausea	19 (26)

Vomiting	9 (12.3)

Diarrhea	38 (52.1)

Headaches	46 (63)

Loss of taste	39 (53.4)

Loss of smell	39 (53.4)

Fatigue	35 (47.9)

Malaise	5 (6.8)

Myalgias or body aches	32 (43.8)

Sore throat	10 (13.7)

Chills	22 (30.1)

Nasal congestion	6 (8.2)

Other	31 (42.5)

**Duration of symptoms in days (median [range])**	14 (1–43)

**Days from symptom onset to SARS-CoV-2 PCR sample collection (median [range])**	6 (0–36)

**Days from positive SARS-CoV-2 PCR to 1-month blood sample collection (median [range])**	32 (14–76)

**Days from symptom onset to 1-month blood sample collection (median [range])**	41 (21–83)

**Hospitalization**	6 (8.2)

**COVID medications**	

Hydroxychloroquine	2 (2.7)

Chloroquine	1 (1.4)

Azithromycin	14 (18.9)

Lopinavir/ritonavir	0 (0)

Remdesivir	0 (0)

Convalescent plasma	0 (0)

None	58 (79.5)

Other	2 (2.7)

**Month 4**	
**Days from positive SARS-CoV-2 PCR to 4-month blood sample collection (median [range])**	122 (102–192)

**Days from symptom onset to 4-month blood sample collection (median [range])**	131 (106–193)

**Any symptom present month 4 visit**	24 (32.9)

Fever	0 (0)

Cough	1 (1.4)

Dyspnea	7 (9.6)

Nausea	1 (1.4)

Vomiting	1 (1.4)

Diarrhea	2 (2.7)

Headaches	1 (1.4)

Loss or altered taste	7 (9.6)

Loss or altered smell	12 (16.4)

Fatigue	9 (12.3)

Forgetfulness/brain fog	8 (11)

Hair loss	5 (6.8)

Other	7 (9.6)

*Joint pain*	3 (4.1)

**Month 7**	
**Days from positive SARS-CoV-2 PCR to 7-month blood sample collection (median [range])**	**216 (191–259)**

**Days from symptom onset to 7-month blood sample collection (median [range])**	225 (194–268)

**Any symptom present month 6 visit**	30 (41.1)

Fever	1 (1.4)

Cough	0 (0)

Dyspnea	6 (8.2)

Nausea	1 (1.4)

Vomiting	0 (0)

Diarrhea	1 (1.4)

Headaches	3 (4.1)

Loss or altered taste	8 (11)

Loss or altered smell	11 (15.1)

Fatigue	13 (17.8)

Forgetfulness/brain fog	12 (16.4)

Hair loss	2 (2.7)

Other	11 (15.1)

*Joint pain*	7 (9.6)

**Extended Data Table 2.** Healthy control characteristics

Variable	Total N= 11 N (%)
**Age (median [range])**	38 (23–53)
**Sex**	
Female	4 (36.4)
Male	7 (63.6)
**Race**	
White	8 (72.7)
Black	1 (9.1)
Asian	1 (9.1)
